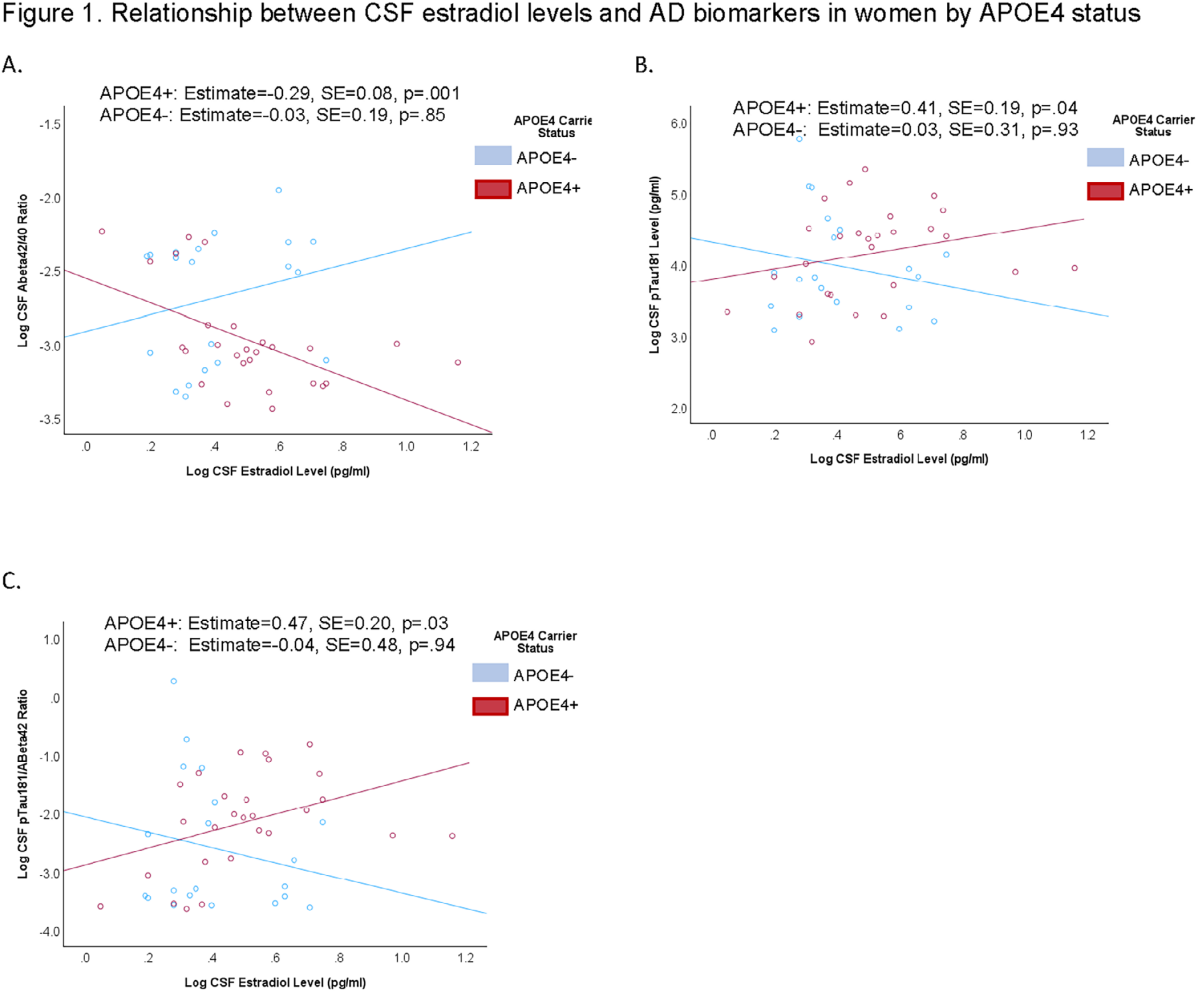# Sex hormones in cerebrospinal fluid relate to Alzheimer's disease biomarkers in a sex and APOE‐dependent manner

**DOI:** 10.1002/alz70856_107385

**Published:** 2026-01-09

**Authors:** Erin E. Sundermann

**Affiliations:** ^1^ University of California, San Diego, La Jolla, CA, USA

## Abstract

**Background:**

Given their neuro‐protective and anti‐inflammatory actions, sex hormones are critical to understanding sex differences in Alzheimer's disease (AD). Conventionally, blood‐derived sex hormones have been examined in relation to AD‐related outcomes with inconsistent results. However, sex hormones are produced in the brain and so cerebrospinal fluid (CSF)‐derived sex hormones may better reflect hormonal effects on AD risk, particularly in postmenopausal women in which systemic levels drastically drop. We measured sex hormones in CSF and examined their relationships with CSF AD biomarkers by sex and APOE4 status.

**Method:**

Sex hormones (estradiol, estrone, total testosterone, and progesterone) were measured using liquid chromatography/tandem mass spectrometry in the CSF of 98 participants (50% female, age range: 48‐92, mean age=72.7, 94% non‐Hispanic white, 48% APOE4 carriers) from the UCSD Alzheimer's Disease Research Center who had CSF AD biomarker data available (Aβ42/40, *p*‐Tau181, *p*‐Tau181/Aβ42). The sample was comprised of 59% cognitively normal, 12% MCI, 24% mild AD, and 5% with unavailable diagnosis. Generalized linear models examined relationships between sex hormone levels and AD biomarkers, and the moderating role of APOE4 status, in sex‐stratified analyses. Age, BMI, hypertension and current/past smoking were included as covariates.

**Result:**

In females, the sex hormone X APOE4 interactions did not reach significance (*p*s≤0.21); however, APOE4‐stratified analyses revealed that higher CSF estradiol levels significantly related to lower Aβ42/40 and higher *p*‐Tau181 and *p*‐Tau181/Aβ42 in female APOE4 carriers only (*n* = 26; *p*s<.05; Figure 1). Regardless of APOE4 status, estrone, testosterone and progesterone did not relate to AD biomarkers in females, and no sex hormones related to AD biomarkers in males.

**Conclusion:**

Higher CSF estradiol levels related to more advanced AD biomarker levels in female APOE4 carriers. No significant associations were observed in male participants. Estrogen promotes APOE expression, and this may amplify the negative effects of the APOE4 allele in females who are more susceptible to these effects. Findings may inform prior reports of a beneficial effect of menopause‐associated hormone therapy on cognitive outcomes in APOE4 non‐carriers only. Additional work is needed to replicate these results in larger samples and to compare CSF‐derived versus plasma‐derived sex hormones association with AD biomarkers.